# Conophylline inhibits high fat diet-induced non-alcoholic fatty liver disease in mice

**DOI:** 10.1371/journal.pone.0210068

**Published:** 2019-01-28

**Authors:** Tomohiko Ohashi, Yukiomi Nakade, Mayu Ibusuki, Rena Kitano, Taeko Yamauchi, Satoshi Kimoto, Tadahisa Inoue, Yuji Kobayashi, Yoshio Sumida, Kiyoaki Ito, Haruhisa Nakao, Kazuo Umezawa, Masashi Yoneda

**Affiliations:** 1 Division of Gastroenterology and Hepatology, Department of Internal Medicine, Aichi Medical University, Nagakute, Aichi, Japan; 2 Department of Molecular Target Medicine, Aichi Medical University, Nagakute, Aichi, Japan; IDIBAPS Biomedical Research Institute, SPAIN

## Abstract

Conophylline (CnP), a vinca alkaloid extracted from the leaves of the tropical plant *Tabernaemontana divaricate*, attenuates hepatic fibrosis in mice. We have previously shown that CnP inhibits non-alcoholic steatohepatitis (NASH) using a methionine-choline-deficient (MCD) diet-fed mouse model. However, little is known about the CnP mediated inhibition of hepatic steatosis in high-fat diet-induced non-alcoholic fatty liver disease (NAFLD) mouse models. CnP (0.5 and 1 μg/g/body weight) was co-administered along with a high-fat diet to male BALB/c mice. After nine weeks of administering the high-fat diet, hepatic steatosis, triglyceride, and hepatic fat metabolism-related markers were examined. Administration of a high-fat diet for 9 weeks was found to induce hepatic steatosis. CnP dose-dependently attenuated the high-fat diet-induced hepatic steatosis. The diet also attenuated hepatic peroxisome proliferator-activated receptor alpha (PPARA) mRNA levels. PPARA is known to be involved in β-oxidation. CnP upregulated the mRNA levels of hepatic PPARA and its target genes, such as carnitine palmitoyl transferase 1 (CPT1) and CPT2, in a dose-dependent manner in the liver. Furthermore, levels of hepatic β-hydroxybutyrate, which is a type of ketone body, were increased by CnP in a dose-dependent manner. Finally, CnP increased the expression of the autophagosomal marker LC3-II and decreased the expression of p62, which are known to be selectively degraded during autophagy. These results indicate that CnP inhibits hepatic steatosis through the stimulation of β-oxidation and autophagy in the liver. Therefore, CnP might prove to be a suitable therapeutic target for NAFLD.

## Introduction

With increase in the westernization of dietary habits, the prevalence of non-alcoholic fatty liver disease (NAFLD) has reached 25% of the world population [1]. NAFLD is considered the hepatic indicium of metabolic syndrome, which is a group of cardiovascular risk factors [2–4]. On the other hand, non-alcoholic steatohepatitis (NASH) which is defined with steatosis, necroinflammation and cytopathic changes, causes liver cirrhosis and lies on the spectrum of NAFLD.[5] Although the pathogenesis of NASH remains to be elucidated, many parallel hits derived from the adipose tissue and the gut are thought to facilitate liver inflammation [6]. Endoplasmic reticulum stress and its related signaling networks, adipocytokines, and innate immunity are emerging as central pathways that regulate the key characteristics of NASH [6]. There is still lack of effective therapy for NASH at present. As a result, it is desirable to identify new therapeutic strategies for the regulation of steatohepatitis [7, 8].

Conophylline (CnP) is a vinca alkaloid, which was initially isolated from the leaves of the tropical plant *Tabernaemontana divaricata* in Malaysia [9]. Later, CnP was isolated from the leaves of *Ervatamia microphylla* as a compound that induces pancreatic β-cell differentiation in cultured cells and animals [10, 11]. The possible mechanism underlying the action of CnP could involve the increase in the mRNA expression levels of pancreas duodenum homeobox-1, neurogenin3, and neuroD/Beta2, which are essential for the differentiation of cultured porcine pancreatic endocrine cells [12]. Oral administration of CnP was found to inhibit pancreatic islet fibrosis in Goto-Kakizaki rats [13]. It also inhibited fibrosis in a chemically induced rat liver cirrhosis model by reducing the activity of α-smooth muscle antigen (αSMA) cells and increasing the production of collagen [14].

We have previously shown that CnP improves steatohepatitis in mice through the downregulation of transforming growth factor-β (TGF-β) and the upregulation of peroxisome proliferator-activated receptor α (PPARA) involved in fatty acid oxidation using a methionine-choline-deficient diet [14, 15]. Although a methionine-choline-deficient diet has been shown to induce steatohepatitis, which is morphologically similar to NASH, it does not lead to body weight gain and obesity [16]. On the other hand, mice on high-fat diets have been used as NAFLD models [17, 18], and little is known about CnP-mediated attenuation of NAFLD in high-fat diet mouse model.

In the present study, we examined whether CnP attenuates NAFLD in BALB/c mice that were fed a high-fat diet. We demonstrated that CnP improves steatosis in mice through the upregulation of PPARA and its downstream targets involved in fatty acid oxidation and autophagy.

## Materials and methods

### Substances and treatments

CnP was originally isolated from the leaves of a Tabernaemontana divaricate plant grown on the Miyako-jima island in Okinawa, Japan. It was not necessary to obtain field permits to collect these plant samples because we purchased the plant leaves from a local company through Japan Tobacco Company. The crude extract was partially purified as described previously [19, 20]. For *in vivo* study, we used the crude CnP preparation II that was extracted and purified as described previously [20]. The high-fat diet 32 (HFD) contained 25% proteins, 29% carbohydrates, and 32% fats (with saturated, monounsaturated, and polyunsaturated fatty acids added at 7, 22, and 4 g/100 g chow, respectively) [21]. HFD has a calorific value of 507 kcal/100 g. The fat-origin calorific rate is 60% of the gross energy.

### Animal model and experimental design

Four-week-old male BALB/c mice were purchased from CLEA Inc. (Tokyo, Japan). After a 1-week acclimatization period on a basal diet (Oriental Yeast), 20 mice were divided into four groups and fed one of the following diets for 9 weeks: (1) control diet (n = 5), (2) HFD (n = 7), (3) HFD with CnP (0.5 μg/g/d body weight per os) (n = 4), or (4) HFD with CnP (1 μg/g/d body weight per os) (n = 4). Doses of CnP were determined in accordance with those used in a previous study [14]. CnP was included in the pellet of HFD as per the energy consumption [22]. All mice were given free access to water and experimental diets. Body weights of the mice in each group were recorded weekly. Protocols regarding the use of mice were approved by the Institutional Animal Care and Use Committee of the Aichi Medical University. The handling of mice was in accordance with the National Institutes of Health “Guide for the Care and Use of Laboratory Animals.” After being fed the experimental diets for 9 weeks, the mice were euthanized by CO_2_ inhalation without fasting. Livers were rapidly excised, and then either fixed in buffered formalin (10%) or frozen in liquid nitrogen and stored at –80 °C. Blood samples were collected from the left ventricle and centrifuged and the serum was stored at –80 °C.

### Serum and tissue biochemical measurements

As described previously [14, 15], serum alanine aminotransferase (ALT) and fasting blood glucose (FBG) levels were determined using commercially available kits (Wako, Osaka, Japan). Serum immunoreactive insulin (IRI) levels were measured using a mouse insulin ELISA kit (Funakoshi, Tokyo, Japan). Stored liver samples (100 mg) were lysed and homogenized in a 2 mL solution containing 150 mM NaCl, 0.1% Triton X-100, and 10 nM Tris using a polytron homogenizer (NS-310E; MicroTech Nichion, Tokyo, Japan) for 1 min. Hepatic triglyceride (TG), free fatty acid (FFA) and β-hydroxybutyrate contents were measured using a triglyceride detection kit (Wako), a free fatty acid detection kit (Wako), and β-hydroxybutyrate assai kit (Cayman chemical, Ann Arbor, MI, USA) respectively. Serum TG, FFA, and cholesterol levels were also measured using a triglyceride detection kit (Wako), free fatty acid detection kit (Wako), and cholesterol detection kit (Wako), respectively.

### Histopathological examination

Five-micrometer-thick sections of liver samples originally fixed in formalin and embedded in paraffin were examined in all experiments as described previously [14, 15]. Hematoxylin-eosin staining was performed to assess hepatic steatosis. Oil Red O staining was performed using a standard technique to examine hepatic fat deposition. Oil red O positive area was quantified in 5 randomly selected fields per section. The percentage of Oil Red O positive area was measured using a computerized image analysis system in Image-Pro Plus version 4.5 (Media Cybernetics, Silver Spring, MD, USA).

### Real-time polymerase chain reaction of liver RNA

As described previously [14, 15], frozen liver tissues were homogenized using the TRIzol reagent (Life Technologies, Tokyo, Japan) and RNA extraction was performed using the RNeasy Mini kit (Qiagen, Tokyo, Japan). The isolated RNA was resuspended in 40 μL of RNase-free water and quantified by spectrophotometry (optical density [OD] 260 and low-mass gel electrophoresis [Invitrogen, Tokyo, Japan]). The total RNA extracted was reverse transcribed to cDNA using a High Capacity cDNA Reverse Transcription kit (Applied Biosystems, Foster City, CA) according to the manufacturer’s instructions. Real-time quantitative PCR was carried out using the ABI StepOne Sequence Detection System (Applied Biosystems) using TaqMan Gene Expression Assays (acyl-coenzyme A oxidase 1 [ACOX1], Mm01246834_m1; autophagy related 7 [Atg7], Mm00512209_m1; apolipoprotein B [apoB], Mm01545150_m1; cluster of differentiation 36 [CD36], Mm00432403_m1; cluster of differentiation 68 [CD68], Mm00839636_g1; carnitine palmitoyltransferase 1 [CPT1], Mm00463960_m1; carnitine palmitoyltransferase 2 [CPT2], Mm00487205_m1; fatty acid synthase [FASN] Mm00662319_m1; sterol regulatory element binding transcription factor 1 [SREBF1], Mm00550338_m1; microsomal triglyceride transfer protein [MTTP], Mm00435015_m1; [PPARA], Mm00440936_m1; sequestosome-1 [SQSTM1], Mm00448491_m1; tumor necrosis factor-α [TNF-α], Mm00443258_m1; Toll like receptor 4 [TLR4], Mm00445273_m1; [TGF-β], Mm00441724_m1; and tissue inhibitor of metalloproteinase 1 [TIMP1], Mm00441818_m1), and the TaqMan Universal PCR Master Mix (Applied Biosystems) according to the manufacturer’s instructions. The detailed protocol used for TaqMan PCR was described in a previous study [23].

### Western blot analysis

Liver tissues were homogenized in the SDS sample buffer, separated in a 10% SDS-polyacrylamide gel, and electro-transferred to nitrocellulose membranes. After blocking with 5% nonfat dry milk in TBST buffer [10 mmol/L Tris-HCl (pH 8.0), 150 mmol/L NaCl, 1% Tween 20], the membranes were probed with anti-microtubule-associated protein light chain 3 (LC3) antibodies (dilution 1:1000, Cell Signaling, Inc., Danvers, MA, USA) and anti-p62 antibody (dilution 1:1000, MBL, Nagoya, Japan) followed by incubation with HRP-conjugated anti-rabbit immunoglobulin G secondary antibodies (1:2000; DAKO Japan, Tokyo, Japan). Antibody binding was then visualized with enhanced chemiluminescence reagent (GE Healthcare, Tokyo, Japan). Protein bands images were obtained using the LAS1000 gel documentation system (Fuji Film, Tokyo, Japan) and were densitometrically analyzed using the Image Gauge software (Fuji Film, Tokyo, Japan).

#### Immunohistochemistry

The immunohistochemical analysis was performed as described previously [14, 15], using formalin-fixed, paraffin-embedded liver sections. After deparaffinization and rehydration in xylene and graded alcohols, endogenous peroxidase was quenched with hydrogen peroxide. Nonspecific binding was blocked with 10% normal goat serum in phosphate buffered saline (PBS) (Wako, Tokyo, Japan). Incubation with anti-LC3 rat antibody (dilution 1:200, Cell Signaling Technology, Denver, MA, USA) and anti-p62 antibody (dilution 1:1000, MBL, Nagoya, Japan) were followed by incubation with goat anti-rabbit IgG secondary antibody (dilution 1:1000, Thermo Fisher Scientific, Waltham, MA, USA). Specimens were observed under a microscope (Keyence BZ-9000, Osaka, Japan). For the semi-quantitative morphometric analysis, the numbers of LC3 and p62 positive cells were calculated in a blinded fashion and averaged for five fields per slide at a 200X magnification by using Image-Pro Plus computerized image analysis system version 4.5 (Media Cybernetics, Silver Spring, MD, USA).

### Statistical analysis

All results were expressed as mean ± standard error (SE). Statistical analyses were performed using analysis of variance (ANOVA). A P value of less than 0.05 was considered statistically significant.

Bonferroni post hoc test was performed to analyze the differences between the multiple groups.

## Results

### Inhibition of hepatic steatosis by conophylline

**Fig 1:**
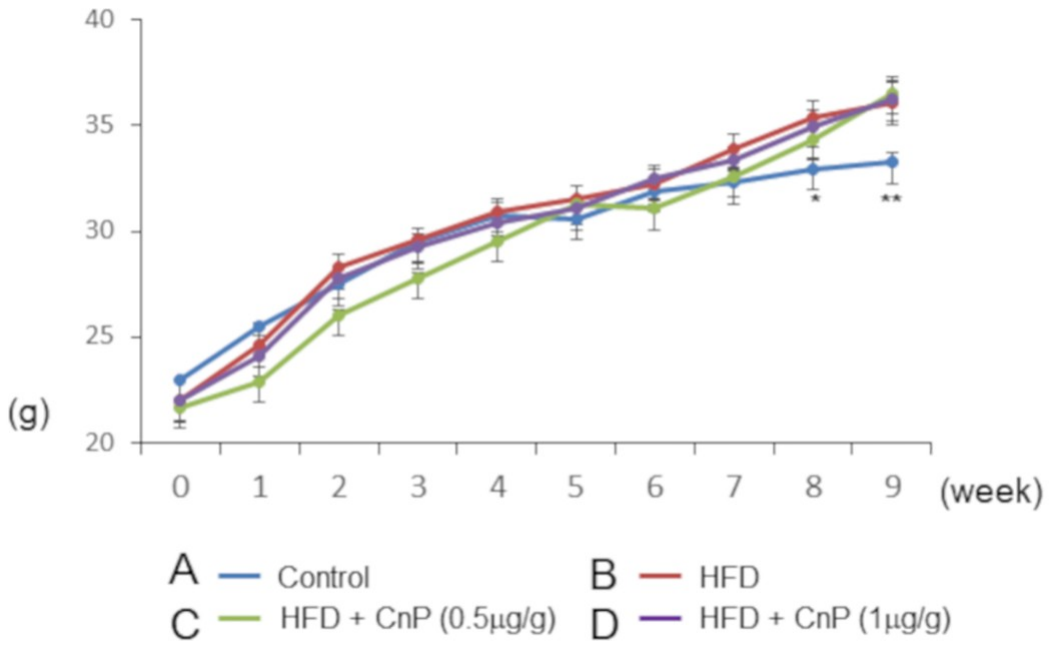
Time course alterations in body weight. Mice were fed with a control diet (Control, n = 5) (A), high-fat diet (HFD, n = 7) (B), HFD with conophylline [HFD + CnP (0.5 μg/g), n = 4] (C) or HFD with conophylline [HFD + CnP (1 μg/g), n = 4] (D). Statistical analysis was performed using ANOVA, and data are expressed as means ± SE (*P < 0.05 for control diet compared to HFD diet; ** P < 0.01 for HFD diet compared to a control diet).

**Fig 2:**
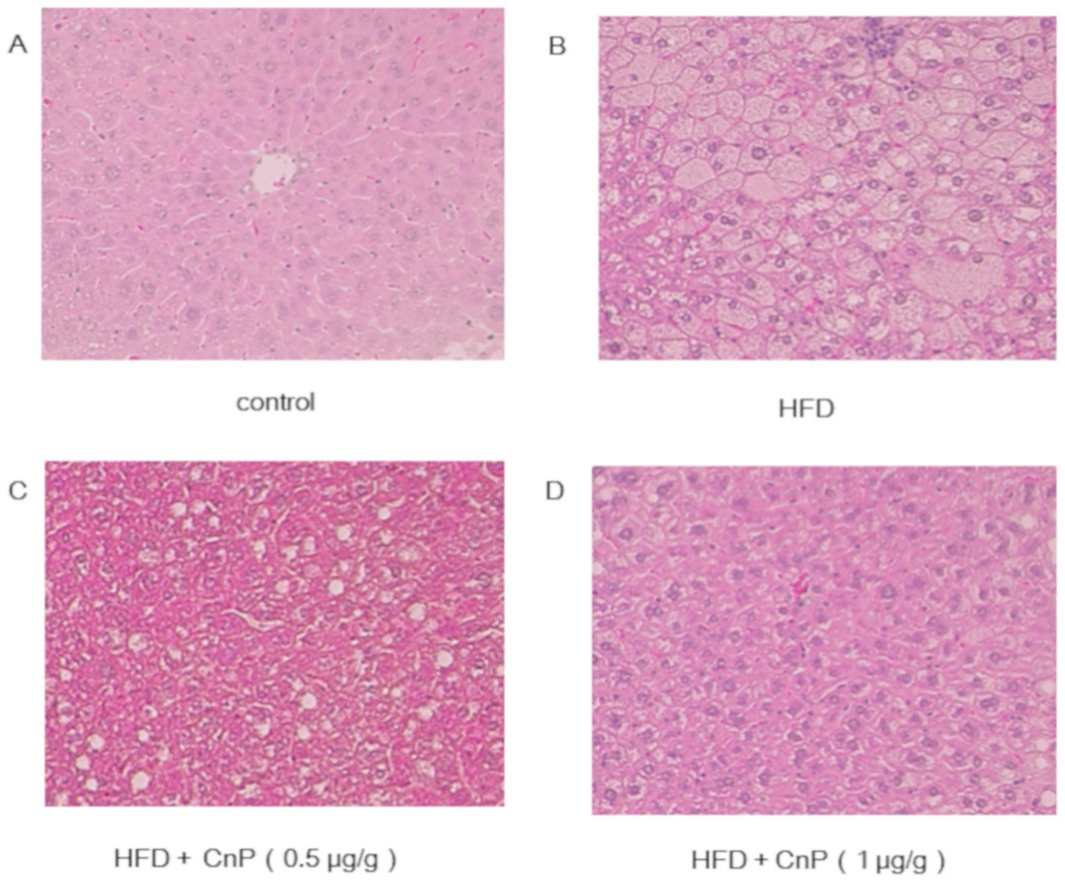
Representative images of the liver tissues stained with hematoxylin-eosin stain. Mice were fed with a control diet (Control, n = 5) (A), high-fat diet (HFD, n = 7) (B), HFD with conophylline [HFD + CnP (0.5 μg/g), n = 4] (C) or HFD with conophylline [HFD + CnP (1 μg/g), n = 4] (D). Original magnification, 100X.

**Fig 3:**
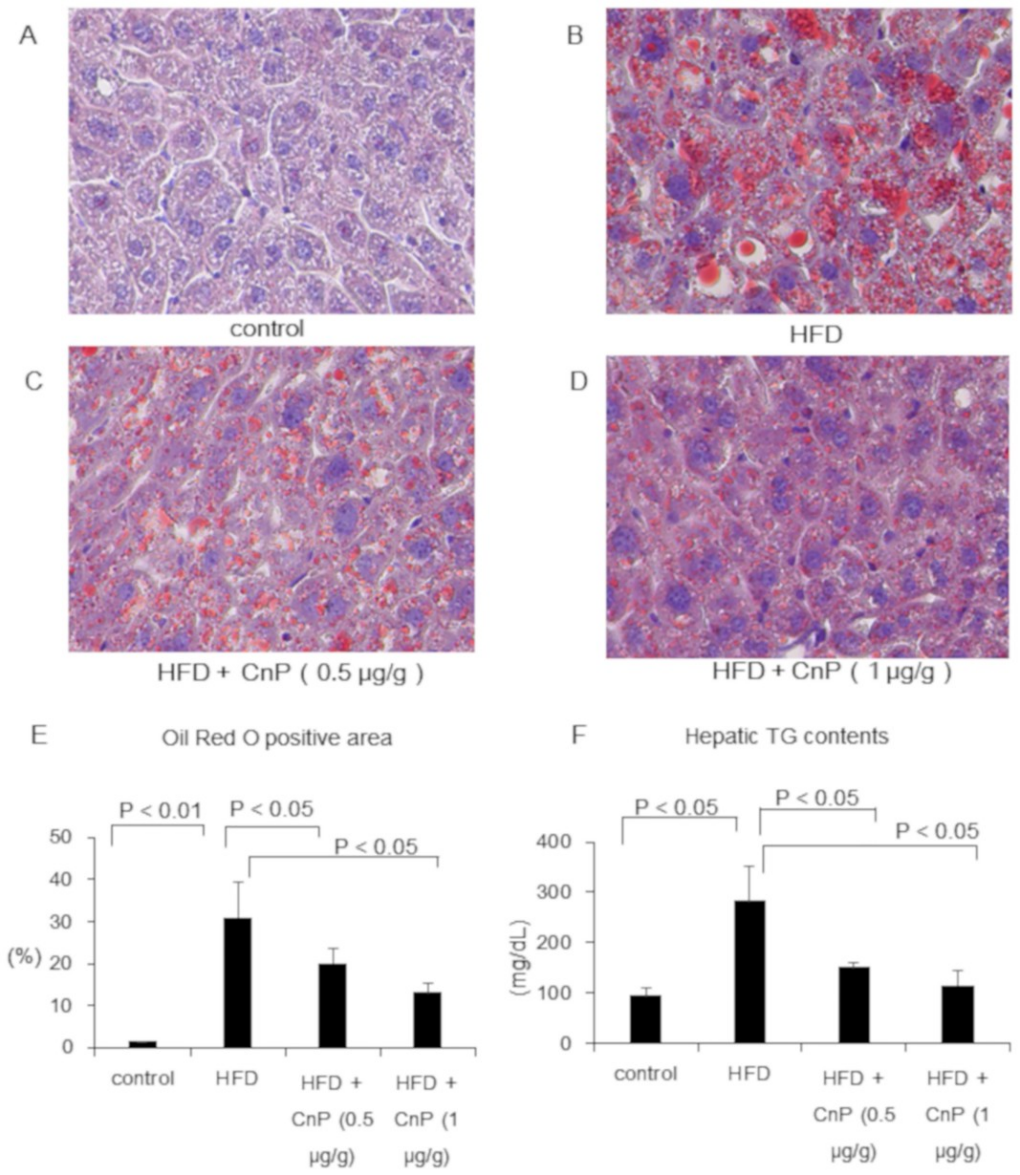
Representative images of the liver stained with Oil Red O and hepatic TG contents. Mice were fed with a control diet (Control, n = 5) (A), high-fat diet (HFD, n = 7) (B), HFD with conophylline [HFD + CnP (0.5 μg/g), n = 4] (C), or HFD with conophylline [HFD + CnP (1 μg/g), n = 4] (D). Original magnification, 200X. (E) Oil red O positive area was quantified in 5 randomly selected fields per section. The percentage of Oil Red O-positive area was measured using a computerized image analysis system with Image-Pro Plus version 4.5 (Media Cybernetics, Silver Spring, MD, USA).(F) Hepatic TG contents (mg/dL) in the respective groups. Statistical analysis was performed using ANOVA, and the data are expressed as means ± SE.

Body weights of HFD-fed mice were significantly increased in comparison to control mice after 7 weeks of the commencement of feeding (Fig 1). HFD diet increased body weight gain, which was not changed upon CnP treatment throughout the experimental period (Fig 1). Mice fed with HFD gained subepididymal fat weight and were unaltered by CnP treatment throughout the experimental period (Table 1). Hematoxylin-eosin staining showed that HFD increases hepatic steatosis, which is attenuated by CnP treatment (Fig 2A–2D). Oil Red O staining also showed that HFD increased the Oil Red O-positive area, which was inhibited by CnP treatment in a dose-dependent manner (Fig 3A–3E). HFD significantly increased hepatic TG contents, which were decreased by CnP treatment in a dose dependent manner (Fig 3F). HFD significantly increased hepatic FFA contents, which decreased upon CnP treatment (Table 2). In contrast, HFD did not alter serum TG levels, which were also not altered by CnP treatment (Table 2). HFD also did not alter serum FFA levels, which decreased upon CnP treatment (Table 2). HFD increased serum cholesterol levels, which were not altered by CnP treatment (Table 2). Compared with control diet-fed mice, mice fed with HFD showed no change in FBG levels, which were also not changed by CnP treatment (Table 1). HFD increased serum IRI levels, which were further augmented by CnP treatment at a dose of 0.5 μg/g. As a result, serum IRI levels of HFD mice upon CnP treatment were significantly higher than that of control. In contrast, serum IRI levels of HFD mice were not further increased upon CnP treatment at a dose of 1μg/g (Table 1).

**Table 1:** Clinical characteristics of mice fed with experimental diets.

Group	n	Body weight gain (g)	Subepididymal fat weight (g)	FBG (mg/dL)	IRI (μg/L)
Control	5	7.8 ± 0.3	0.41 ± 0.03	197 ± 19	0.3 ± 0.1
HFD	7	12.2 ± 1.1^a^	0.63 ± 0.08	215 ± 12	1.3 ± 0.3
HFD + CnP (0.5 μg/g)	4	13.3 ± 0.9^a^	0.53 ± 0.05	198 ± 12	3.1 ± 0.8^a^
HFD + CnP (1 μg/g)	4	11.7 ± 0.7^a^	0.69 ± 0.06	205 ± 11	1.6 ± 0.3

HFD, high-fat diet; CnP, conophylline; FBG, fasting blood glucose; IRI, serum immunoreactive insulin

Data were analyzed using ANOVA. Values represent means ± SE.

^a^ P < 0.05 for each group compared to control.

**Table 2:** Hepatic and serum TG and FFA contents in mice fed with experimental diets.

Group	n	Serum TG (mg/dL)	Hepatic FFA (mEq/L)	Serum FFA (mEq/L)	Serum cholesterol (mg/dL)
Control	5	118 ± 7.46	0.42 ± 0.02	1.02 ± 0.05	82.1 ± 4.8
HFD	7	118 ± 5.48	0.69 ± 0.03^a^	1.05 ± 0.09	156 ± 28
HFD + CnP (0.5 μg/g)	4	109 ± 7.91	0.61 ± 0.05	0.88 ± 0.04	140 ± 6.3
HFD + CnP (1 μg/g)	4	107 ± 10.37	0.60 ± 0.05	0.82 ± 0.05	150 ± 3.8

HFD, high-fat diet; CnP, conophylline; Data were analyzed using ANOVA.

Values represent means ± SE. The overall P values for steatosis, activity grade, and fibrosis were less than 0.05.

^a^ P < 0.05 for control compared to HFD

### Activation of the expression of genes related to energy consumption by conophylline

**Fig 4:**
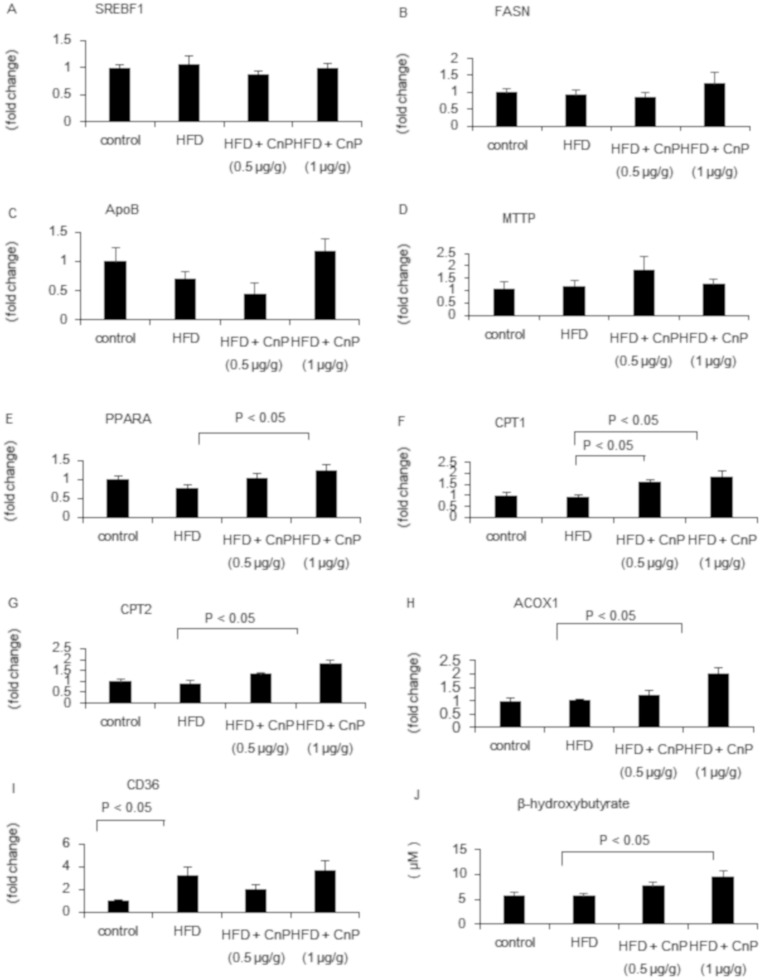
Evaluation of hepatic lipid metabolism-related genes. Mice were fed a control diet, high-fat diet (HFD), HFD with conophylline (HFD + CnP, 0.5 μg/g), or HFD with conophylline (HFD + CnP, 1 μg/g). Relative mRNA expression levels of SREBF1 (A), FASN (B), ApoB (C), MTTP (D), PPARA (E), CPT1 (F), CPT2 (G), ACOX1 (H), and CD36 (I) were evaluated. Hepatic β-hydroxybutyrate contents were evaluated using ELISA (J). Statistical analysis was performed using ANOVA, and the data are expressed as means ± SE.

HFD did not alter hepatic SREBF1, FASN, ApoB, MTTP, PPARA, CPT1, CPT2, and ACOX1 mRNA levels (Fig 4A–4H). In contrast, HFD significantly increased CD36 mRNA levels (Fig 4I). Hepatic SREBF1, FASN, ApoB, MTTP, and CD36 mRNA levels were not altered by CnP treatment in HFD-fed mice (Fig 4A, 4B, 4C, 4D and 4I). In contrast, hepatic PPARA, CPT1, CPT2, and ACOX1 mRNA levels were dose-dependently increased upon CnP treatment in HFD-fed mice (Fig 4E–4I). Hepatic β-hydroxybutyrate levels were dose-dependently increased upon CnP treatment in HFD-fed mice (Fig 4J).

### Enhancement of autophagy by the regulation of Atg7 and SQSTM1 by conophylline

**Fig 5:**
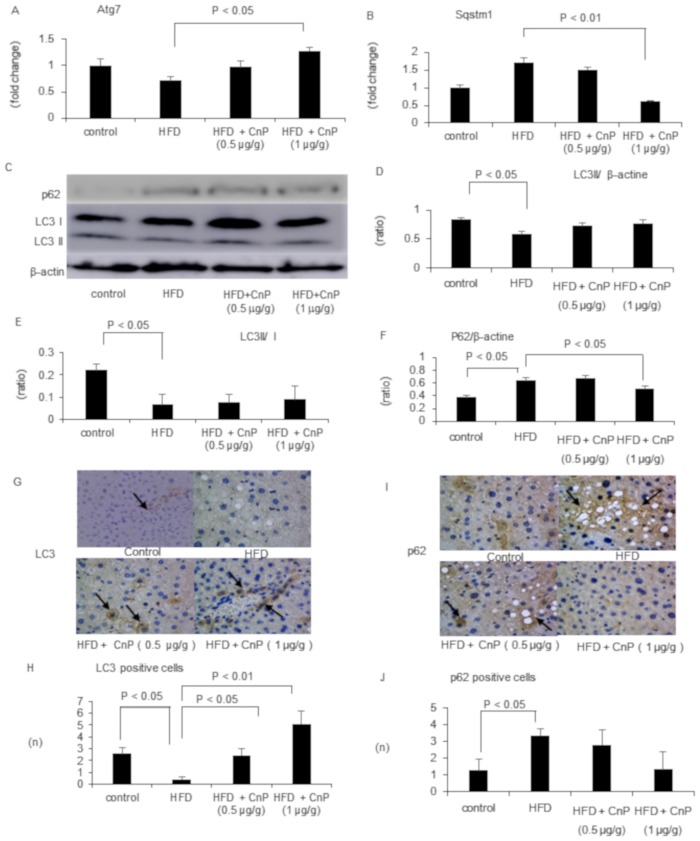
Evaluation of hepatic autophagy-related parameters. Mice were fed with a control diet, high-fat diet (HFD), HFD with conophylline (HFD + CnP, 0.5 μg/g), or HFD with conophylline (HFD + CnP, 1 μg/g). Relative mRNA expression of Atg7 (A) and SQSTM1 (B). Western blot autoradiograph images (C) and levels (D, E) of LC3 and p62 proteins on autophagy in mice. The ratio of LC3II/I (F). Immunohistochemical staining of LC3 proteins of mice liver. Arrows indicate hepatic LC3 positive cells (G). Quantitative analysis of changes in the immunohistochemical positive LC3 cells in the respective groups (H). Immunohistochemical staining of p62 proteins of mice liver. Arrows indicate hepatic p62 positive cells (I). Quantitative analysis of changes in the immunohistochemical positive p62 cells in the respective groups (J). Results show the representative pictures of mice (original magnification, 100X). Statistical analysis was performed using ANOVA, and the data are expressed as means ± SE.

Activation of autophagy might be helpful for decreasing fat in the cells and tissues. CnP was reported to activate autophagy in rat pheochromocytoma PC12 cells [24]. Therefore, we studied its effect on autophagy in mice. HFD inhibited Atg7 mRNA levels, which were increased in a dose-dependent manner upon CnP treatment (Fig 5A). On the other hand, HFD increased SQSTM1 mRNA levels, which were significantly decreased by CnP treatment at a dose of 1 μg/g (Fig 5B). Western blot analysis showed that HFD significantly decreased LC3II protein expression (Fig 5C and 5D). Elevated levels of LC3II were observed following CnP treatment (Fig 5C and 5D). The ratio of LC3II/I was also significantly decreased by HFD supplementation, which also tended to increase upon CnP treatment (Fig 5E). HFD significantly increased p62 protein expression, which was significantly attenuated upon CnP treatment (Fig 5F). HFD also significantly reduced the number of LC3 positive cells, which dose-dependently increased upon CnP treatment (Fig 5G and 5H). HFD significantly increased the number of p62 positive cells, which tended to decrease upon CnP treatment at a dose of 1.0 μg/g (Fig 5I and 5J)

### Hepatic inflammatory response by conophylline

**Fig 6:**
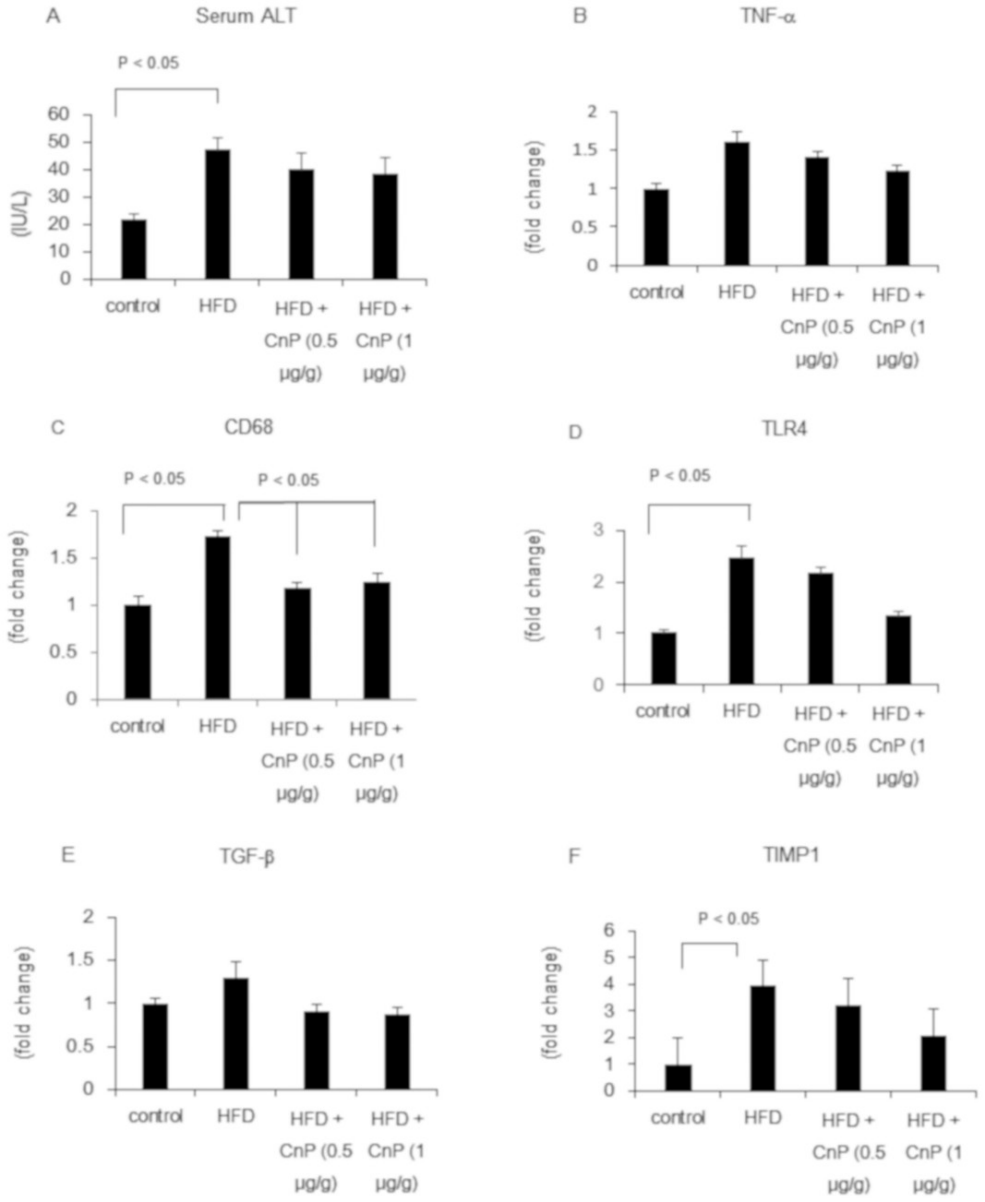
Evaluation of hepatic inflammation-related parameters. Mice were fed with a control diet, high-fat diet (HFD), HFD with conophylline (HFD + CnP, 0.5 μg/g), or HFD with conophylline (HFD + CnP, 1 μg/g). Serum alanine aminotransferase (ALT) levels (A). Relative mRNA expression levels of TNF-α (B), CD68 (C), TLR4 (D), TGF-β (E), and TIMP1 (F) were evaluated in the liver. Statistical analysis was performed using ANOVA, and the data are expressed as means ± SE.

HFD significantly increased serum ALT and hepatic TNF-α mRNA levels, which were reduced upon CnP treatment (Fig 6A and 6B). HFD significantly increased CD68 mRNA levels, which were significantly reduced following CnP treatment (Fig 6C). HFD significantly increased TLR4 mRNA levels, which tended to decrease upon CnP treatment (Fig 6D). HFD increased hepatic TGF-β mRNA levels, which decreased following CnP treatment (Fig 6E). HFD significantly increased hepatic TIMP-1 mRNA levels, which decreased following CnP treatment (Fig 6F).

## Discussion

In the current study, we investigated whether CnP inhibits HFD-induced hepatic steatosis in a NAFLD mouse model. We demonstrated that administration of CnP reduced HFD-induced hepatic steatosis that was measured as hepatic TG contents. Furthermore, HFD-induced increase in the hepatic inflammation-related markers, which were further attenuated following CnP administration.

Animal models and dietary approaches are useful tools to simulate NAFLD observed in humans. A methionine-choline-deficient diet is widely used and highly reproducible model of accelerated hepatic steatosis, inflammation, and fibrosis in the context of NASH, despite the associated loss of body weight and insulin resistance [25]. Compared to MCD diet, HFD-based models are relatively time consuming; however, the use of HFD led to the development of preclinical models mimicking the full spectrum of the metabolic and histological features of human NAFLD, including obesity, insulin resistance, and liver inflammation and fibrosis[26]. Although high-fat diet feeding for nine weeks induced distinct liver steatosis, along with significant increase in subepididymal fat weight and IRI levels [21, 22, 27], serum ALT and AST levels are not significantly increased until 34–36 weeks after the commencement of diet feeding [28]. Therefore, long-term HFD studies will be required to determine the extent of CnP mediated attenuation of hepatic inflammation and fibrosis.

A previous report showed that CnP stimulates pancreatic β-cell differentiation, and dose dependently increases serum IRI levels in rat. In the present study, though CnP increased serum IRI levels at a dose of 0.5 μg/g, it did not increase serum IRI levels significantly at a dose of 1 μg/g. The reason for the lack of significant increase in serum IRI levels by CnP at a dose of 1 μg/g remains to be determined. Maximal effective doses of CnP on IRI secretion in the rat might be differ from those in the mice. The strain difference might affect the CnP-induced serum IRI secretion.

Concerns have been raised regarding the fidelity of hepatic fat metabolism, FFA influx, hepatic *de novo* lipogenesis, β-oxidation, and very low density lipoprotein (VLDL) excretion in the NASH model [25, 29, 30]. The fatty acid transport protein CD36 is reported to be involved in FFA uptake in the liver [25, 31]. VLDL has been reported to play an important role in hepatic TG excretion [7]. ApoB and MTTP are proteins related to the production of VLDL and excretion of TG from hepatocytes [32]. A previous report indicated that the hepatic influx of FFA and impairment of VLDL secretion contributes to an important novel mechanism in MCD-induced hepatic steatosis [25]. In human NASH, it has been reported that the predominant source of hepatic lipid is hepatic uptake through inappropriate peripheral lipolysis [33]. In the present study, hepatic FFA was significantly increased in mice fed with HFD, and CnP attenuated HFD-induced FFA augmentation in the liver. CD36 mRNA levels, which were significantly increased in the HFD mouse model, were not altered following CnP treatment. HFD did not increase hepatic SREBF1 and FASN that are related to hepatic *de novo* lipogenesis, and they were not affected by CnP. HFD did not alter hepatic apoB and MTTP mRNA levels, which did not change significantly following CnP treatment. These results indicated that CnP-induced reduction of steatosis was not involved in FFA influx, hepatic *de novo* lipogenesis, and VLDL excretion.

Hepatic ACOX1, CPT1, and CPT2 are involved in β-oxidation in the liver [34–36]. HFD did not significantly affect hepatic ACOX1, CPT1, and CPT2 mRNA levels, which were further increased upon CnP treatment. PPARA is expressed in the liver and is involved in hepatic lipid metabolism [37]. It has been reported that PPARA gene expression was correlated with severity and histological treatment response in NASH [38]. The administration of PPARA agonists have been shown to attenuate HFD-induced hepatic TG accumulation [39] and up-regulate β-oxidation enzymes, thereby reducing hepatic TG in mice [40]. In the present study, we showed that hepatic PPARA mRNA levels were dose-dependently increased following CnP treatment. Furthermore, hepatic β-hydroxybutyrate levels were increased by HFD. These results led to the hypothesis that CnP reduced HFD-induced FFA augmentation through the stimulation of hepatic β-oxidation in the liver.

Although HFD-induced inflammation and the expression of fibrosis related genes were increased, distinct hepatic inflammation and fibrosis were not observed in the present study. Assessment of hepatic inflammation-related genes expression in the liver revealed that HFD tended to augment hepatic TNF-α, TGF-β, and TIMP-1 mRNA levels, and significantly augmented hepatic CD68 and TLR4 mRNA levels. It has been demonstrated that CnP inhibits TGF-β-induced apoptosis in rat hepatoma cells [41]. We demonstrated that CnP decreased hepatic CD68, TLR4, TGF-β, and TIMP-1 mRNA levels, indicating that CnP attenuated hepatic inflammation signaling. Previous reports have indicated that reduced TGF-β signaling attenuates steatohepatitis [42]. Therefore, CnP-induced attenuation of TGF-β signaling might reduce steatosis and inflammation signaling.

Next, we examined whether CnP-induced inhibition of hepatic steatosis was involved in autophagy. Autophagy is initiated by the formation of a small membrane particle, called the autophagosome. The autophagosome is generated through various cell signaling cascades involving autophagy-related genes and LC3 [43]. The interrelationship between β-oxidation and autophagy has been reported [44]. A knockdown of the autophagy gene Atg5 in hepatocytes increased TG levels and inhibited β-oxidation [44]. This finding indicates that the inhibition of autophagy decreases β-oxidation in the liver. In the present study, hepatic Atg7 mRNA levels, LC3 protein levels, and immunohistochemical expression were attenuated by HFD; and CnP restored Atg7 mRNA levels, LC3 protein levels, and immunohistochemical expression at a dose of 1 μg/g. Furthermore, hepatic SQSTM1 mRNA levels and p62 immnuohistochemical positive cells, which are selectively degraded by autophagy, were increased by HFD. On the other hand, CnP reduced SQSTM1 mRNA levels and decreased p62 protein positive cells. These results indicated that HFD impaired hepatic autophagy, which was improved following CnP administration.

In conclusion, the data obtained in the present study suggest that CnP attenuates steatosis through the stimulation of PPARA, CPT1, and CPT2-related β-oxidation in the liver. Furthermore, CnP stimulates hepatic autophagy resulting in a decrease in hepatic lipotoxicity and an increase in the expression of inflammation-related genes. CnP could accordingly be considered as a therapeutic option to prevent hepatic steatosis in NAFLD.

## Supporting information

S1 Fig Western blot imaging for β-actine. (JPG)

S2 Fig Western blot imaging for LC3. (TIF)

S3 Fig Western blot imaging for p62. (TIF)
