# Supplementary figures and images for: Conophylline inhibits high fat diet-induced non-alcoholic fatty liver disease in mice
Source: PLoS One. 2019 Jan 28;14(1):e0210068. doi: 10.1371/journal.pone.0210068 (PMC6349312; doi:10.1371/journal.pone.0210068)

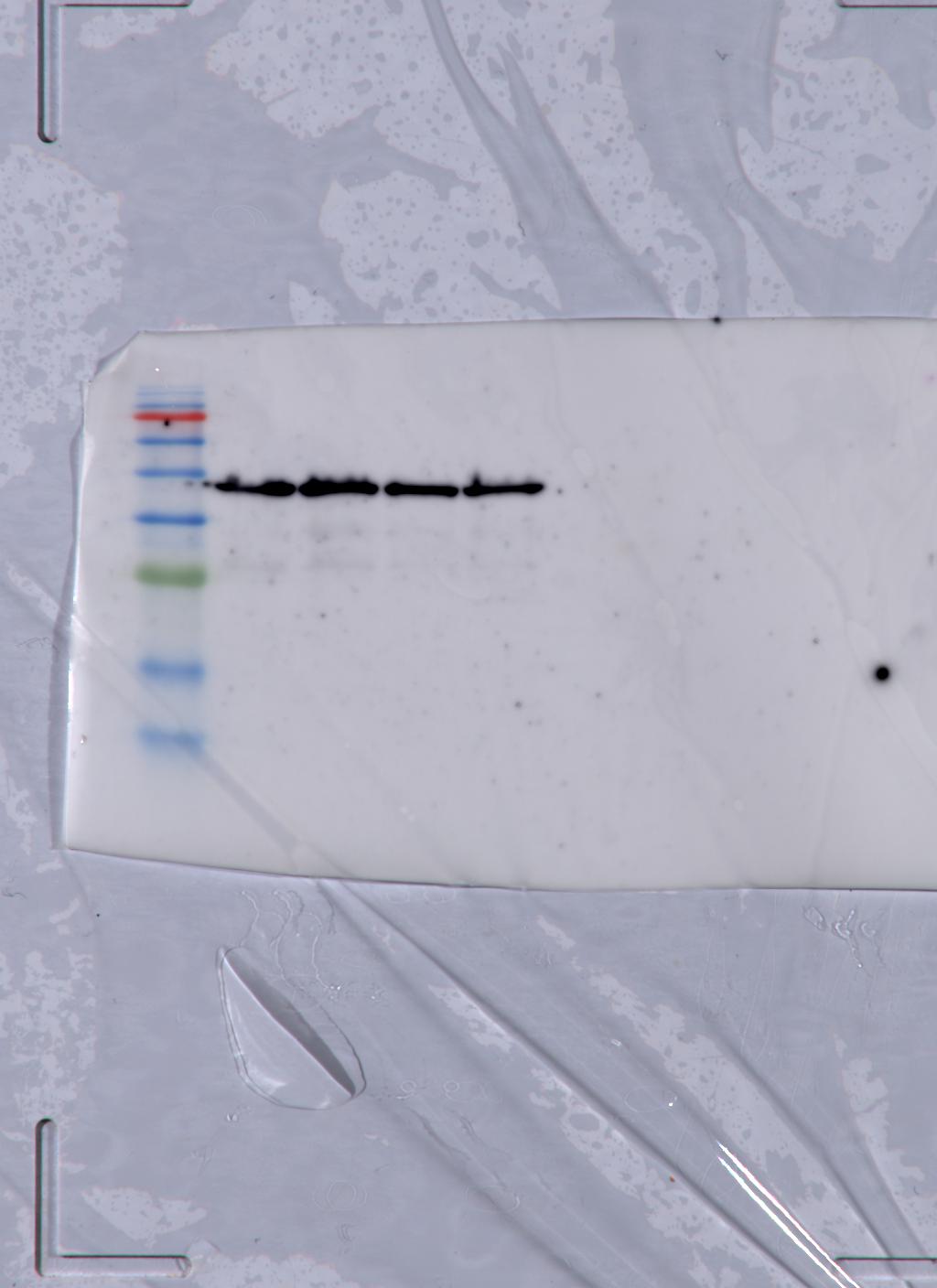

Supplement: S1 Fig — (JPG) [file pone.0210068.s001.jpg]

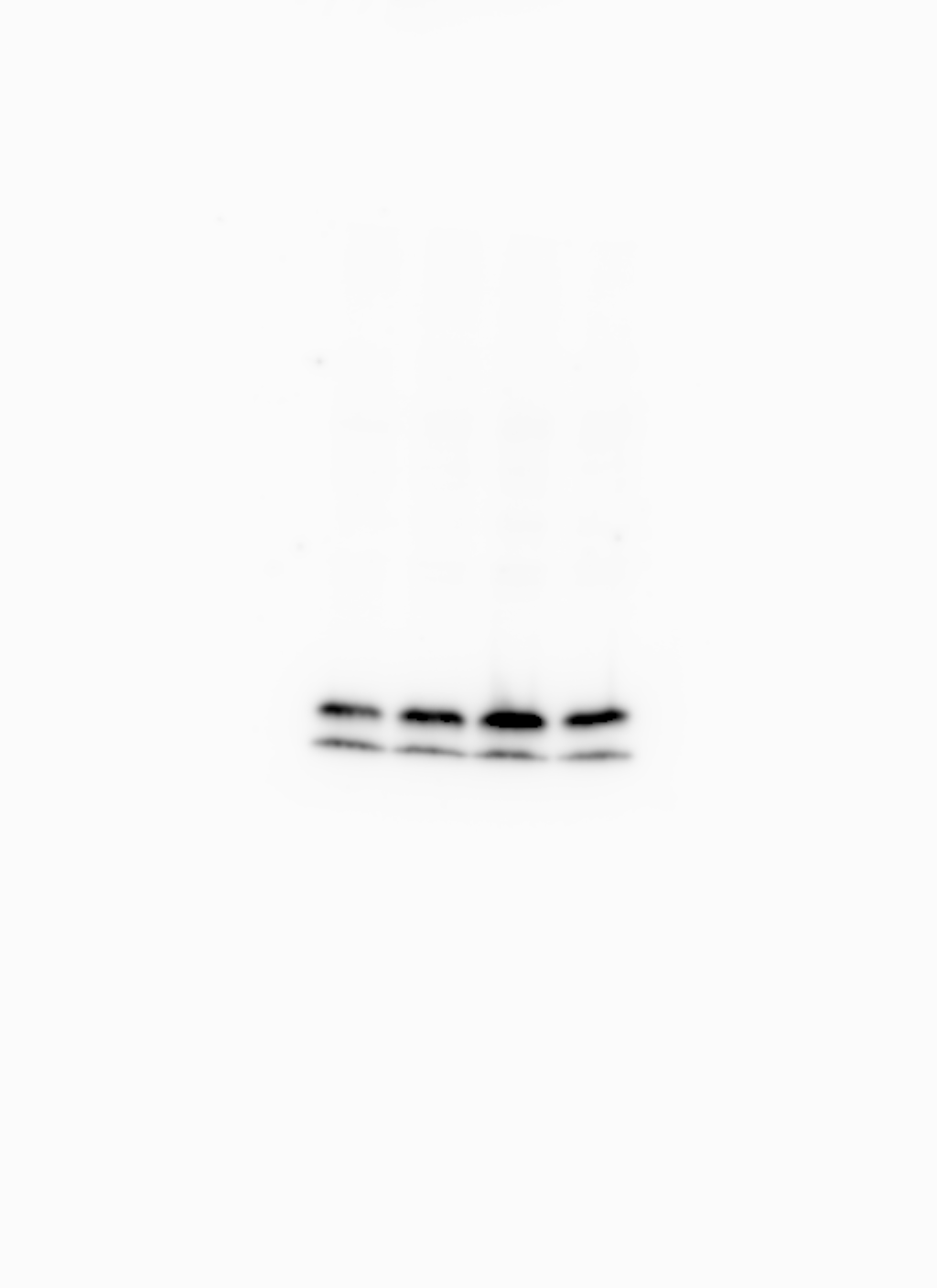

Supplement: S2 Fig — (TIF) [file pone.0210068.s002.tif]

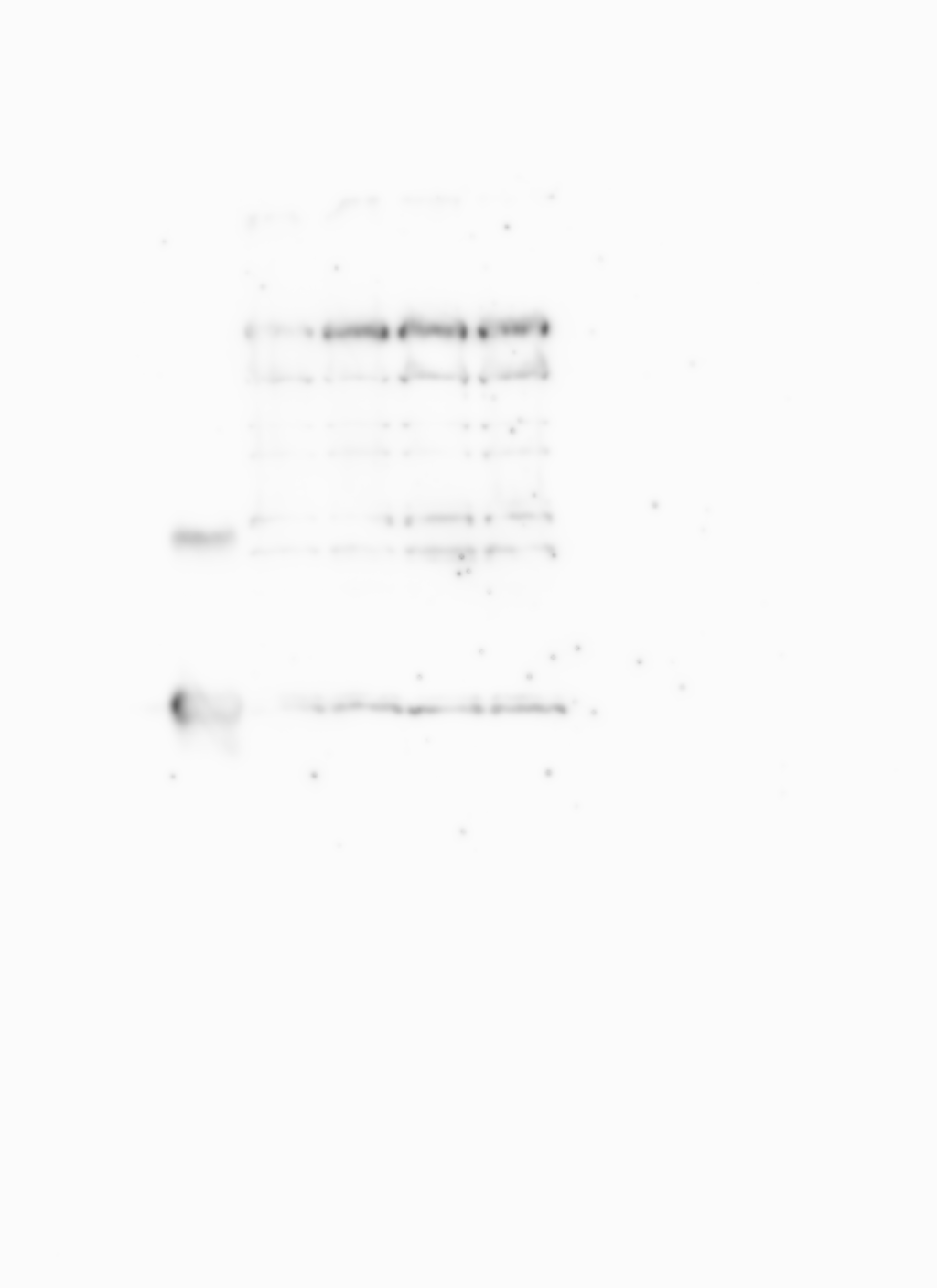

Supplement: S3 Fig — (TIF) [file pone.0210068.s003.tif]
